# Theoretical and Numerical Analysis of Active Switching for Narrow-Band Thermal Emission with Graphene Ribbon Metasurface

**DOI:** 10.3390/s21206738

**Published:** 2021-10-11

**Authors:** Kyohei Yada, Takashi Shimojo, Hideyuki Okada, Atsushi Sakurai

**Affiliations:** 1 Graduate School of Science and Technology, Niigata University, 8050, Ikarashi 2-no-cho, Niigata 950-2181, Japan; glatt_ta87im65@yahoo.co.jp (K.Y.); st_chiroru@yahoo.co.jp (T.S.); f18b085g@mail.cc.niigata-u.ac.jp (H.O.); 2 Department of Mechanical and Production Engineering, Niigata University, 8050, Ikarashi 2-no-cho, Niigata 950-2181, Japan

**Keywords:** metasurface, thermal emission, graphene plasmon, electromagnetic simulation, Fabry-Perot resonance, equivalent circuit model

## Abstract

Components smaller than the wavelength of electromagnetic waves are called meta-atoms. Thermal emission can be controlled by an artificial structure in which these meta-atoms are arranged on the surface. This artificial structure is called a metasurface, and its optical properties are determined by the materials and shapes of the meta-atoms. However, optical devices may require active control of thermal emission. In the present study, we theoretically and numerically analyze a wavelength-selective emitter using a graphene ribbon metasurface. The graphene ribbon metasurface consists of a graphene ribbon array, potassium bromide thin film, and silver substrate. The geometric parameters of the graphene metasurface are determined based on an equivalent circuit model that agrees well with the results of the electromagnetic field analysis (rigorous coupled-wave analysis). The proposed emitter causes impedance matching depending on the conductivity of the graphene ribbon in a very narrow wavelength range. The conductivity of graphene can be actively controlled by the gate voltage. Therefore, the proposed emitters may realize near-perfect emission with a high quality factor and active controllable switching for various wavelengths. In addition, the quality factor can be changed by adjusting the electron mobility of graphene. The proposed emitter can be used for optical devices such as thermophotovoltaic systems and biosensing.

## 1. Introduction

Thermal emission is a spontaneous and continuous photon emission from the thermal reservoir [1]. Therefore, it was difficult to realize active switching (i.e., on/off switching) of thermal emission without mechanical shutter. In addition, thermal emission is a broadband light because it originates from the fluctuating current in a material; however, narrow-band thermal emission is a promising technology for the improvement of thermal devices [2].

A meta-atom is a component smaller than the wavelength of an electromagnetic wave. An artificial material that realizes functions that are difficult with natural materials by arranging meta-atoms on the surface is called a metasurface. In recent years, nano/micro-scale artificial materials called metasurfaces were used to control thermal emission. Controlling light with metasurfaces has the possibility to realize active switching of narrow-band thermal emission. Active switching of narrow-band thermal emission is a key technology with applications in thermophotovoltaic systems [3,4], infrared heaters [5], biosensing [6,7,8], microbolometers [9,10], imaging [11], and optical communications [12].

Normally, the optical properties of metasurfaces may be determined by their geometrical shape [13,14,15]. Therefore, thermal emission cannot be actively controlled by conventional metasurfaces. To solve this problem, we focus on graphene, which is one of the carbon allotropes, and has a honeycomb structure where carbon atoms are bonded two-dimensionally [16]. One of the characteristics of graphene is that its electrical conductivity changes when a gate voltage is applied. Thus, active switching of thermal emission may be possible without changing the shape of the structure using graphene metasurfaces [17,18].

In the present study, we propose a graphene ribbon metasurface to design a device for realizing narrow-band emission peak at the target wavelength and active wavelength control. This structure can be designed based on an equivalent circuit model. We computationally demonstrate that the designed graphene ribbon metasurface can exhibit near-perfect narrow-band thermal emission under active switching using electromagnetic wave analysis. In addition, the peak wavelength and intensity of thermal emission can be controlled by adjusting the Fermi energy of graphene.

## 2. Materials and Methods

**Figure 1:**
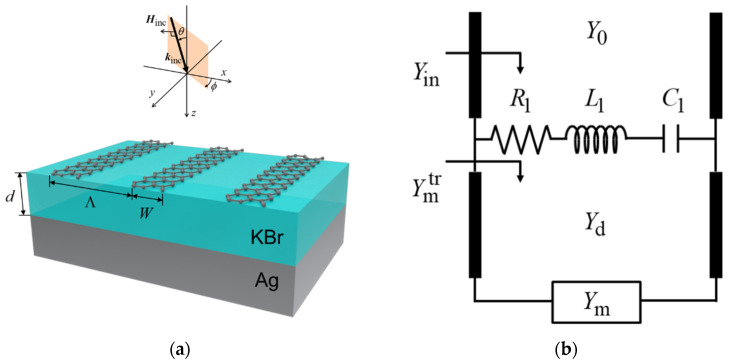
(**a**) Schematic of graphene ribbon metasurface. This structure consists of Ag substrate, KBr layer, and graphene ribbon layer. The period is Λ [µm], graphene ribbon width is *W* [µm], and dielectric thickness is *d* [µm]. Transverse magnetic wave is used as the incident wave. (**b**) Equivalent circuit model based on graphene plasmon.

Figure 1a shows the schematic of the proposed graphene ribbon metasurface emitter. It consists of a graphene ribbon array, a dielectric thin film, and a metallic substrate. Such ribbon-shaped graphene structures can be feasible via top-down structuring such as plasma CVD [19]. The graphene ribbon is periodic in the *x*-direction and extended infinitely in the *y*-direction. The metallic substrate was sufficiently thick. This structure has period Λ [µm], graphene ribbon width *W* [µm], and dielectric thickness *d* [µm]. The optical conductivity of graphene *σ*_g_ [S] is given as [20]

(1) $$\sigma_{g}=\ln\frac{2\left|E_{F}\right|-\left(\omega -i\tau^{-1}\right)\hbar }{2\left|E_{F}\right|+\left(\omega -i\tau^{-1}\right)\hbar }+\frac{-ie^{2}k_{B}T}{\pi \hbar^{2}\left(\omega -i\tau^{-1}\right)}\left\{\frac{E_{F}}{k_{B}T}+2\ln\left(e^{-E_{F}/k_{B}T}+1\right)\right\},$$

where *E*_F_ [eV] is the Fermi energy of graphene, *ω* [rad/s] is the angular frequency in vacuum, *τ* [ps] is the relaxation time of graphene, *ħ* [m^2^kg/s] is the reduced Planck constant, *e* [C] is the electron charge, *k*_B_ [m^2^kg/s^2^K] is the Boltzmann constant, and *T* [K] is the temperature of graphene. The Fermi energy of graphene can be tuned by applying a gate voltage.

The permittivity of graphene *ε*_g_ is given as [21]

(2) $$\varepsilon_{g}=\frac{i\sigma_{g}}{\varepsilon_{0}\omega \Delta },$$

where *ε*_0_ [F/m] is the permittivity of vacuum and *∆* [nm] is the thickness of the graphene.

The equivalent circuit model is useful for designing graphene ribbon metasurfaces and elucidating the underlying mechanism of emissivity enhancement [22]. Figure 1b shows the equivalent circuit model corresponding to Figure 1a. *Y*_0_ [S] is the admittance of free space, *Y*_in_ [S] is the admittance of graphene ribbon metasurface, *Y*_d_ [S] is the admittance of the dielectric layer, *Y*_m_ [S] is the admittance of the metallic substrate, and $Y_{m}^{\mathrm{tr}}$ [S] is the admittance of structure below graphene ribbon. *R*_1_ [Ω], *L*_1_ [H], and *C*_1_ [F] are the resistance, inductance, and capacitance, respectively, corresponding to the first-order mode of graphene plasmon. In this equivalent circuit model, the influence of higher-order modes of graphene plasmon is negligible compared to its first-order mode [23]. The mechanism of emissivity enhancement in this equivalent circuit model is based on the impedance matching theory. A reflected wave is canceled under the impedance matching condition between the graphene ribbon metasurface and vacuum, and thus, nearly perfect emission can be achieved. Since admittance is the reciprocal of impedance, a nearly perfect emission can be obtained when the admittance of the graphene ribbon metasurface is equal to that of the vacuum ($Y_{\mathrm{in}}=Y_{0}$).

Here, *Y*_in_ and *Y*_0_ are given as follows:

(3) $$Y_{\mathrm{in}}=Y_{m}^{\mathrm{tr}}+Y_{G},$$

(4) $$Y_{G}=\frac{1}{R_{1}+i\omega L_{1}+\frac{1}{i\omega C_{1}}},$$

(5) $$Y_{m}^{\mathrm{tr}}=Y_{d}\frac{Y_{m}+iY_{d}\tan\left(\omega nd/c_{0}\right)}{Y_{d}+iY_{m}\tan\left(\omega nd/c_{0}\right)},$$

(6) $$Y_{0}=1/\eta_{0},$$

where *Y*_G_ [S] is the admittance of graphene, *n* is the refractive index of the dielectric layer, *c*_0_ [m/s] is the speed of light in vacuum, and *η*_0_ [Ω] is the impedance of the vacuum (=120*π*). Each coefficient representing the first-order mode of graphene plasmon is given by the following equations in the wavelength range, where the first term on the right side of Equation (1) may be negligible.

(7) $$R_{1}=\frac{\Lambda }{S_{1}^{2}}\frac{\pi \hbar^{2}}{e^{2}E_{F}\tau },$$

(8) $$L_{1}=\frac{\Lambda }{S_{1}^{2}}\frac{\pi \hbar^{2}}{e^{2}E_{F}}=\tau R_{1},$$

(9) $$C_{1}=\frac{S_{1}^{2}}{\Lambda }\frac{2W\varepsilon_{eff}}{\pi r_{1}},$$

(10) $$S_{1}^{2}≅\frac{8}{9}W,$$

where *ε_eff_* is the average relative permittivity of the upper and lower media of graphene, and in this structure $\varepsilon_{eff}=\varepsilon_{0}(1+n^{2})/2$. *r*_1_ is a function of the fill factor and takes a value corresponding to *W*/Λ [22]. It is derived from the surface current density in the graphene plasmon first-order mode obtained using perturbation theory. In this case, the non-perturbative term is the surface current density when light is incident on a single graphene ribbon. The perturbation term is the effect of adjacent ribbons on the surface current density when the ribbons are arranged in a periodic array.

Thus, the conditional expression for admittance matching for a target wavelength *λ*_t_ [µm] is given as

(11) $$R_{1}=\eta_{0},$$

(12) $$L_{1}C_{1}=1/{\left(2\pi f_{t}\right)}^{2},$$

(13) $$\omega_{t}nd/c_{0}=\pi /2,$$

(14) $$Y_{m}\to \infty ,$$

Here, $f_{t}=c_{0}/\lambda_{t}$ [Hz] is the target frequency, and $\omega_{t}=2\pi f_{t}$ [rad/s] is the target angle frequency. The absorptivity predicted by the equivalent circuit model used in this design is given as:

(15) $$A\left(\lambda \right)=1-{\left|\frac{Y_{\mathrm{in}}\left(\lambda \right)-Y_{0}}{Y_{\mathrm{in}}\left(\lambda \right)+Y_{0}}\right|}^{2}.$$

Since the silver (Ag) substrate is opaque, spectral emissivity (*ε_λ_*) can be calculated from the Kirchhoff’s law, i.e., $\varepsilon_{\lambda }=A_{\lambda }$.

Structural admittance α=Re(Yin)η0 and $\beta =nR_{1}^{2}/8\eta_{0}f_{t}L_{1}$ are defined for the real and imaginary parts, respectively. These parameters are useful for determining the bandwidth of the emitter. *α* indicates the value of the real part of the structural admittance at the target wavelength. When *β* is close to unity, the bandwidth becomes narrower and the emissivity increases. As *α* increases from unity, the bandwidth becomes broader and the emissivity decreases. *β* indicates the slope of the imaginary part of the structural admittance at the target wavelength. When *β* is closer to zero, the bandwidth becomes narrower. As *β* increases from zero, the band becomes broader. Therefore, α=1 and β→0 are appropriate for the optimized design of a narrow-band thermal emitter.

The undetermined structural parameters are calculated using the above equations. According to $Y_{\mathrm{in}}=Y_{0}$, Equations (6) and (11) and the definition of *α*,

(16) Re(Yin)=1/R1=α/η0.

Rearranging Equation (13) gives the form:

(17) $$d=\frac{c_{0}}{4nf_{t}}.$$

If it is desirable that the slope of the structural admittance imaginary part is 0,

(18) ddλIm(Yin)=0.

The admittance imaginary part below graphene and the admittance imaginary part of graphene are expressed from Equations (5), (12)–(14) and (17) in the following.

(19) Im(Ymtr)|f≈ft≅πn2η0(fft−1),

(20) Im(YG)|f≈ft≅−2πL1R12f(f2−ft2).

Differentiating Equations (19) and (20) and substituting them into Equations (3) and (18), lead to

(21) $$\frac{\pi n}{2\eta_{0}f_{t}}-\frac{4\pi L_{1}}{R_{1}^{2}}=0.$$

Define *β* as follows:

(22) $$\beta =\frac{nR_{1}^{2}}{8\eta_{0}f_{t}L_{1}}.$$

Substituting Equations (8) and (16) into the Equation (22), lead to

(23) $$\pi =\frac{n}{8\alpha \beta f_{t}}.$$

According to the relation between relaxation time and Fermi energy in graphene,

(24) $$E_{F}=\pi ev_{F}^{2}/\mu .$$

where $v_{F}=10^{6}$ [m/s] is the Fermi velocity of graphene, *µ* [m^2^/Vs] is the electron mobility of graphene. According to Equations (7) and (10),

(25) $$\frac{W}{\Lambda }=\frac{9\pi \hbar^{2}}{8e^{2}E_{F}\tau R_{1}}.$$

According to Equations (8), (9) and (12),

(26) $$W=\frac{r_{1}e^{2}E_{F}}{2\hbar^{2}\omega_{t}^{2}\varepsilon_{eff}}.$$

Λ is determined by Equations (25) and (26). Moreover, there are two restrictions in determining the structural parameters: (1) the graphene ribbon width must not exceed the period; (2) because of the effectiveness of the equivalent circuit model, the period must not exceed the wavelength. Due to the above restrictions,

(27) $$W<\Lambda s_{1},$$

(28) $$\Lambda <\frac{\lambda_{0}}{n}s_{2},$$

where *s*_1_ and *s*_2_ are safety factor parameters, and $s_{1}=0.9$ and $s_{2}=0.8$ are employed for the calculation.

Based on the equivalent circuit model, narrow-band emitters at target wavelengths *λ*_t_ = 6, 8, and 10 µm were designed. A potassium bromide (KBr) layer was used as the dielectric layer, and an Ag substrate was used as the metallic substrate. Here, the phenomenon can be simplified if the refractive index of the dielectric layer is constant. In addition, unintended emission appears when the dielectric layer has the extinction coefficient. Therefore, KBr was used for the dielectric layer, which has a substantially constant refractive index and almost no extinction coefficient in the near-infrared region. The refractive index and dielectric function of KBr was obtained from the tabulated data from Palik’s databook [24]. The dielectric function of Ag was obtained using a Drude model [1]: The permittivity of graphene *ε*_Ag_ is given as $\varepsilon_{\mathrm{Ag}}=\varepsilon_{\infty }-\omega_{p}^{2}/\left(\omega \left(\omega +i\gamma \right)\right)$ with a high-frequency dielectric constant *ε*_∞_ = 3.40, plasma frequency $\omega_{p}=1.39\times 10^{16}$ rad/s, and scattering rate $\gamma =2.70\times 10^{13}$ rad/s. The temperature of graphene was set to be *T* = 300 K, the electron mobility of graphene is *µ* = 2.0 m^2^/Vs, and the structural parameters are determined using the equivalent circuit model. The rigorous couple-wave analysis (RCWA) method [25], which is a semi-analytical method, is employed as an electromagnetic wave analysis. In the RCWA method, the structure is treated as a grid with a uniform dielectric constant distribution in the depth direction. First, the electromagnetic waves in each layer are expressed by Fourier expansion, and the general solution of the electromagnetic field that can exist in the layers is obtained. Next, the solution of the electromagnetic field in the entire region can be obtained by imposing continuous conditions of the electromagnetic field at the boundary of each layer. As a result, the distribution of electromagnetic fields and diffraction efficiency can be calculated. The diffraction order was set to 200, and only transverse magnetic waves were perpendicularly incident on the surface. Note that transverse electric waves were not considered. In the case of transverse magnetic waves, the incident wavevector does not have *y*-component. Therefore, the incident wavevector can be expressed as $k_{\mathrm{inc}}=k_{x}\hat{x}+k_{z}\hat{z}=k_{0}\sin\theta \hat{x}+k_{0}\cos\theta \hat{z}$, where *k*_0_ is the wavevector in vacuum and *θ* is the incident angle. The surface roughness was not considered in the RCWA calculations in this study. However, previous studies have shown that the effect of surface roughness is significant in plasmonic absorbers [26,27]. Although the present calculations dealt with ideal conditions, our future works can deal with this problem by considering periodic structures that approximate the surface roughness in the RCWA calculations. To evaluate the performance of the designed structures, the following quality factor was employed:

(29) $$\begin{array}{cc} Q=\frac{f_{t}}{f_{2}-f_{1}} & \left(f_{1}<f_{t}<f_{2}\right), \end{array}$$

where *f*_1_ [Hz] and *f*_2_ [Hz] are the frequencies at which the emissivity is half the peak emission value on the low-frequency side and high-frequency side of the peak frequency, respectively.

## 3. Results and Discussion

**Figure 2:**
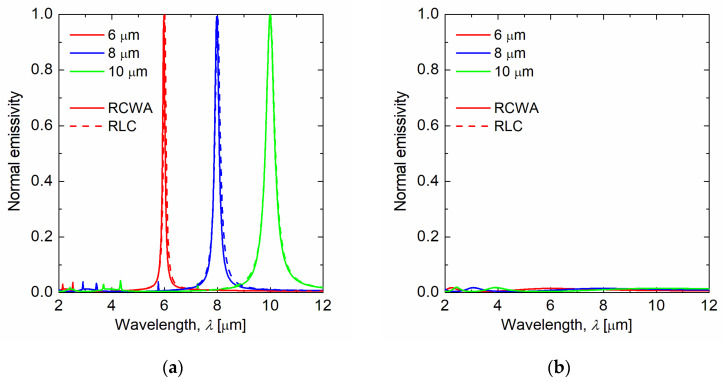
Normal emissivity spectrum for RCWA (colored solid line) and the equivalent circuit model circuit model (colored dashed line) of (**a**) switch-on state and (**b**) switch-off state. Target wavelengths are 6, 8, and 10 µm, and their Fermi energies of switch-on state are 1.64, 1.48, and 1.37 eV, respectively. Their Fermi energies of switch-off state are 0.01 eV.

Figure 2 represents the spectral normal emissivity calculated by the RCWA method (solid line) and the equivalent circuit model (dashed line). Table 1 shows the structural parameters designed for each target wavelength. As shown in Figure 2, the multiple wavelength-selective thermal emissions could be successfully obtained by the RCWA analysis and the equivalent circuit model, and their results agreed well at each target wavelength. Furthermore, the emissivity with *E*_F_ = 0.01 eV is nearly zero in each test case, which implies that active thermal emission switching was also successfully achieved. Therefore, the equivalent circuit theory is significantly effective for designing the graphene ribbon metasurface.

**Figure 3:**
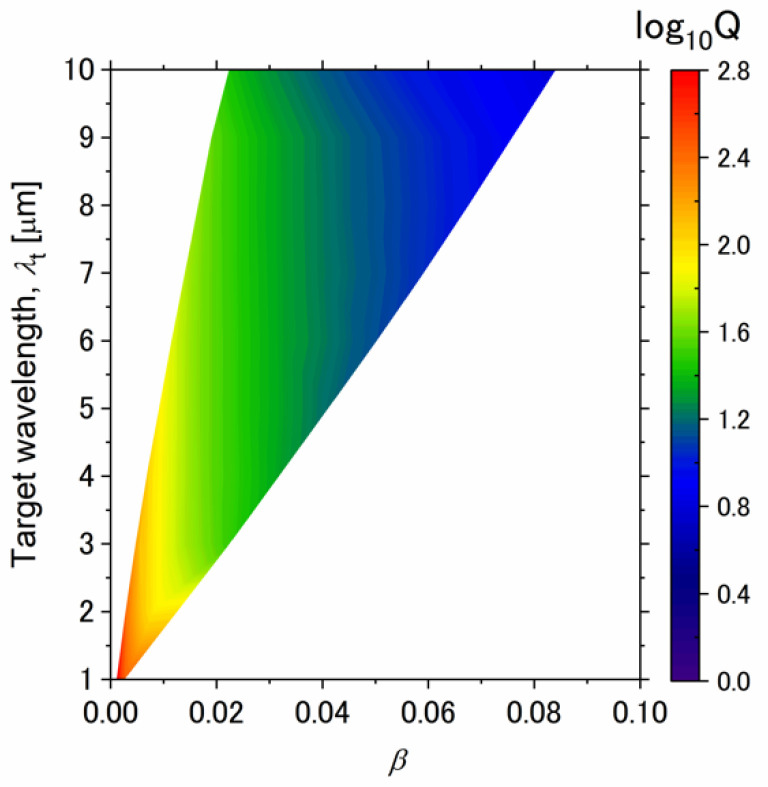
Quality factor for different target wavelengths, and bandwidth parameter *β*. Normal emissivity is calculated from the equivalent circuit model to obtain the quality factor.

Next, we investigated the relationship between the quality factor and the parameter *β*. Table 1 shows the quality factor of each emission spectrum in Figure 2. The quality factor increases as the target wavelength becomes shorter. Figure 3 shows the contour plot of the quality factor with varying target wavelengths and *β*. The white region indicates absence of data because the constraint conditions in Equations (27) and (28) are not satisfied. The quality factor depends only on *β* and is independent of the target wavelengths. Therefore, the design is performed using the smallest *β* among the *β* satisfying Equations (27) and (28). As the target wavelength increases, the minimum value of *β* increases due to the constraint of Equation (28). Therefore, the minimum value of *β* increases as the target wavelength increases, and the quality factor decreases.

**Figure 4:**
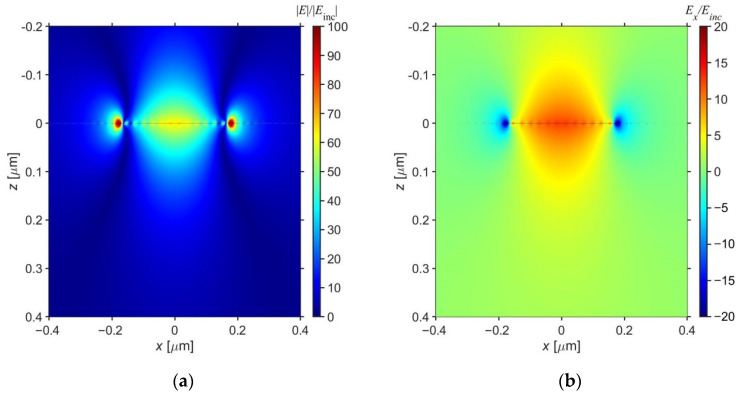
Contour plots of (**a**) Electric field intensity and (**b**) *x*-component of electric field contours around graphene ribbon at the target wavelength *λ*_t_ = 10 µm.

**Figure 5:**
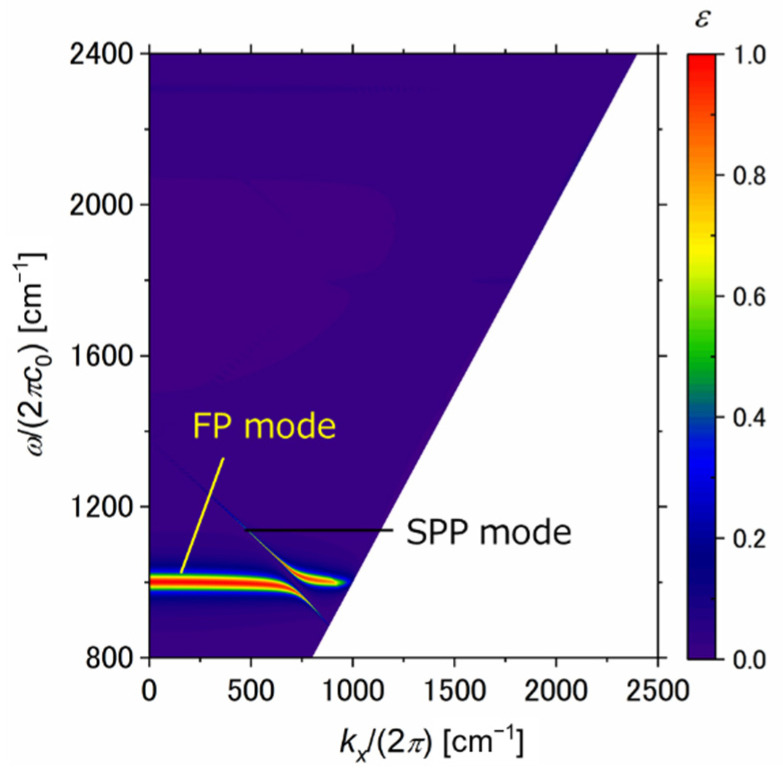
Emissivity contours in terms of the wavevector and *x*-component of wavevector for the graphene ribbon metasurface. The horizontal mode is the FP mode, and the tilted mode is the SPP mode.

In addition, the electric field distribution is shown to investigate the mechanism of the emissivity peak. Figure 4a is a contour diagram of electric field distribution and Figure 4b of *x* component of the electric field from the *x*-*z* plane around the graphene ribbon at the peak wavelength of 10 µm. Figure 5 shows that the electric field is enhanced at the center and edges of the graphene ribbon. Therefore, graphene plasmons are excited in the graphene ribbon [28]. Furthermore, this structure forms an asymmetric FP cavity in which a dielectric layer with a low refractive index is sandwiched between a graphene ribbon array and a metal substrate. In this structure, the excitation of graphene plasmons causes FP resonance [29]. Here, the admittance of the graphene ribbon in the equivalent circuit model is based on the first-order mode of graphene plasmon. Therefore, it supports the excitation of graphene plasmons in Figure 5. As a result, in the proposed structure, the excitation of graphene plasmons in the asymmetric FP cavity causes FP resonance and increases emissivity. The phenomenon can be explained as follows: The emissivity enhancement may be caused by graphene plasmon [22]. When the frequency of the incident photons matches the first localized mode of the graphene ribbons, some photons are absorbed, and others are reflected and transmitted. The transmitted photons are reflected by the metallic substrate, some of which change the phase and exit the structure, resulting in destructive interference. In addition, since the metallic substrate is sufficiently thick, transmission does not occur. Therefore, the incident waves are completely absorbed by the graphene ribbons. On the other hand, when *E*_F_ = 0.01 eV, the normal emissivity is almost zero at any wavelength. This is because graphene plasmon cannot be excited in this situation. Hence, the active switching of emissivity becomes possible by adjusting the Fermi energy in the proposed structure.

Moreover, the dispersion relation is shown in Figure 5 to confirm that the asymmetric FP resonance is excited in the proposed structure. Figure 5 shows that the emission curve is divided into two peaks when the wavevector is increased. This phenomenon is consistent with the characteristics of asymmetric FP resonance [29]. In addition, the division of this emission peak is due to Rabi splitting analogues [29,30,31]. Rabi splitting analogues are a phenomenon in which when two energy levels are combined, a new split energy state appears due to the coupling between the two energy levels and the new eigenstate. The Fabry–Perot mode and surface plasmon polariton (SPP) mode of graphene ribbon are excited in the proposed structure. Therefore, Rabi splitting analogues occur when these modes are coupled. The horizontal mode indicates the FP mode, and the tilted mode indicates the SPP mode in Figure 5.

**Figure 6:**
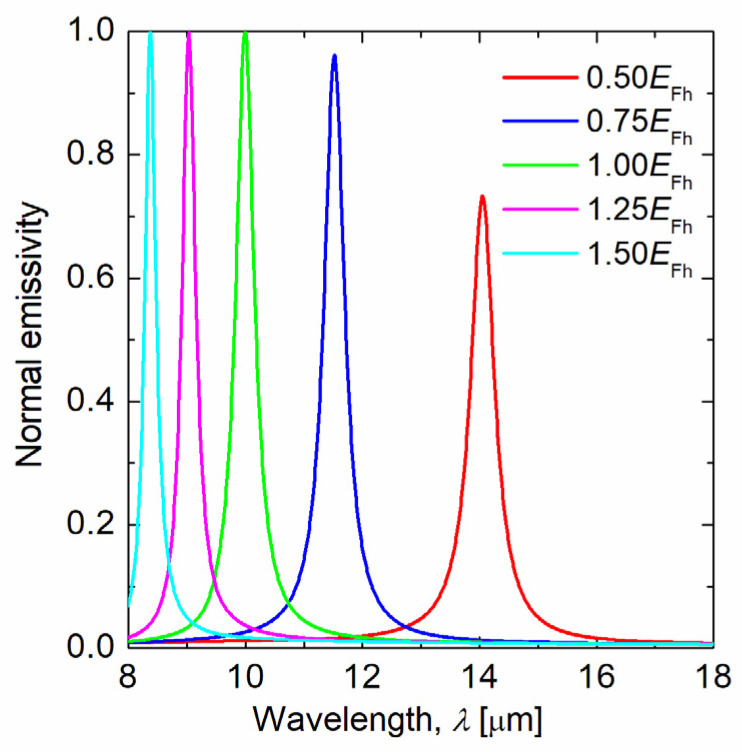
Normal emissivity spectrum for graphene ribbon metasurface with different Fermi energies of graphene. The target wavelength is 10 µm, and the Fermi energy of graphene is 0.5–1.5 times of *E*_F_ = 1.37 eV.

To investigate the effect of Fermi energy, normal emissivity spectra at several Fermi energies were calculated, as shown in Figure 6. In this calculation, the structural parameters of the graphene ribbon metasurface were the same as the test case in which the target wavelength was *λ*_t_ = 10 µm, and the Fermi energy of the graphene was 0.5–1.5 times *E*_F_ = 1.37 eV. As the Fermi energy was reduced, the peak wavelength of the emissivity shifted to a longer wavelength. This is because the resonance wavelength of the graphene plasmon is red-shifted as the Fermi energy decreases. Moreover, as the Fermi energy decreases, the intensity of the emissivity peak decreases. This can be explained using the equivalent circuit model. As the Fermi energy decreases, the conductivity of graphene decreases. Thereby, the resistance and reactance of graphene, and the admittance of graphene, increase. As a result, emissivity decreases because impedance matching is less likely to occur. Since the proposed structure can change the peak wavelength of emissivity by adjusting the Fermi energy of graphene, it can also be used for wavelength control of the thermal emitter without changing the structure. It is important to note that if the Fermi energy is too high for the experiment, the substrate might be destroyed. In this study, we have determined the Fermi energy of graphene based on previous studies with numerical simulations [32,33,34], thus confirming its theoretical performance.

**Figure 7:**
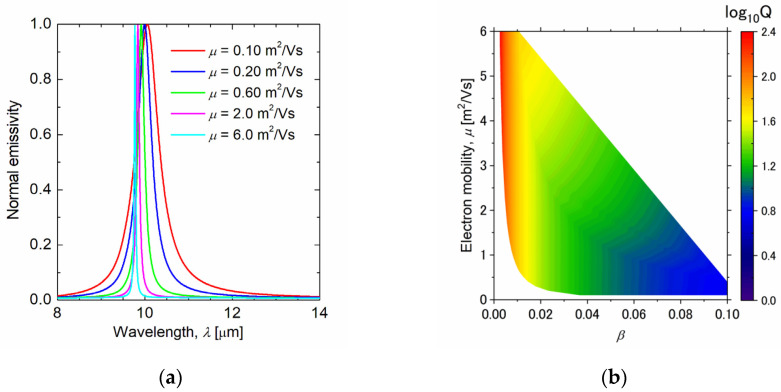
(**a**) Normal emissivity spectrum for graphene ribbon metasurface with different electron mobilities of graphene. The target wavelength is 10 µm, and the electron mobilities of graphene are *µ* = 0.10, 0.20, 0.60, 2.0, and 6.0 m^2^/Vs. (**b**) Quality factor for different mobilities of graphene, and bandwidth parameter *β*. Normal emissivity is calculated from RLC circuit model to obtain the quality factor.

To investigate the effect of changes in the electron mobility of graphene on the structure, the emission spectra at several electron mobilities were calculated, as shown in Figure 7a. Table 2 shows the structural parameters and quality factors for each spectrum. These results show that the quality factor decreases as the electron mobility of graphene decreases. The reason for this can be understood from Figure 7b. Figure 7b shows the quality factor of the emissivity peak of the structure designed by determining the electron mobility of graphene and *β*. The region without data is the region that does not satisfy Equations (27) and (28). The quality factor depends only on *β* and hardly on the electron mobility of graphene (Figure 7b). Similar to the relationship between the target wavelength and the quality factor, the minimum value of *β* increases owing to the constraint of Equation (28) as the electron mobility of graphene increases. Therefore, *β* increases as the electron mobility of graphene increases, and the quality factor decreases. Since the electron mobility of graphene varies depending on the fabrication method, it is important to select the fabrication method according to the desired bandwidth.

**Figure 8:**
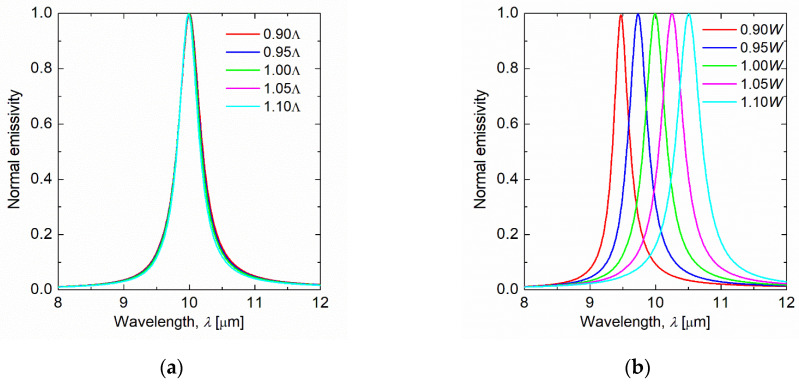

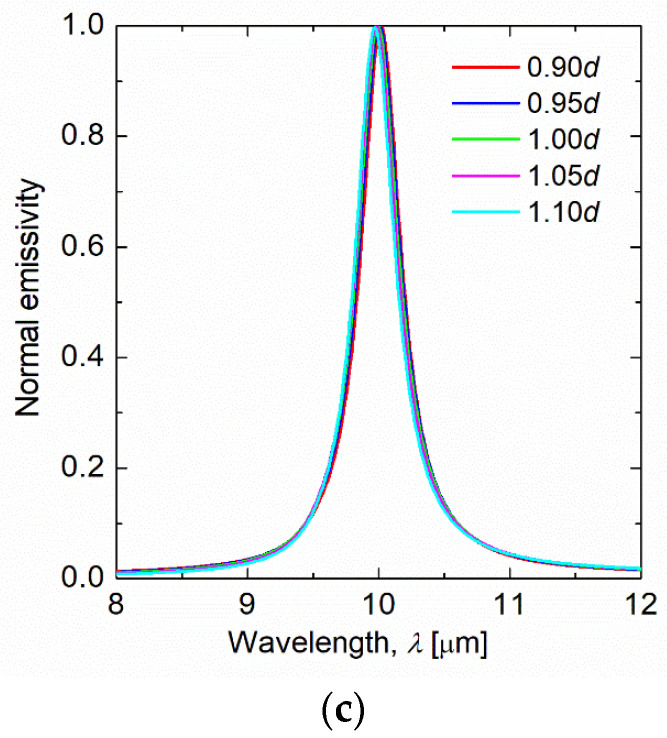
Normal emissivity spectrum for graphene ribbon metasurface with different period, graphene ribbon width, and dielectric thickness. (**a**) The target wavelength is 10 µm, and the period is 0.9–1.1 times of Λ = 5.23 µm. (**b**) The target wavelength is 10 µm, and the graphene ribbon width is 0.9–1.1 times of *W* = 0.355 µm. (**c**) The target wavelength is 10 µm, and the dielectric thickness is 0.9–1.1 times of *d* = 1.64 µm.

In the proposed structure, there are many parameters that can affect the emissivity. Therefore, Figure 8 shows the emissivity spectrum when the period, graphene ribbon width, and dielectric thickness are changed. In this calculation, the structural parameters of the graphene ribbon metasurface were the same as the test case in which the target wavelength was *λ*_t_ = 10 µm. In Figure 8a, the period was 0.9–1.1 times Λ = 5.23 µm, in Figure 8b, the graphene ribbon width was 0.9–1.1 times *W* = 0.355 µm, and in Figure 8c, the dielectric thickness was 0.9–1.1 times *d* = 1.64 µm. Figure 8a,c show that the normal emissivity is nearly independent of the period and dielectric thickness, respectively. However, Figure 8b shows that the peak wavelength of the emissivity shifts to a longer wavelength as the graphene ribbon width is increased. This is because graphene plasmons are excited at the ends of the graphene ribbon (Figure 4), and the resonance wavelength shifts to the longer wavelength as the graphene ribbon width increases.

## 4. Conclusions

In summary, we proposed a graphene ribbon metasurface for active thermal emission switching. Near perfect thermal emission at the targeted wavelengths could be achieved by adjusting the structural parameters, and emissivity switching was achieved by adjusting the Fermi energy of graphene in those structures. In addition, the proposed structure can be easily designed using an equivalent circuit model based on the impedance matching theory. FP cavities are formed in the proposed structure, and graphene plasmons cause an asymmetric FP resonance. The wavelength and intensity of resonance can be changed by adjusting the Fermi energy of graphene. Furthermore, the quality factor may also be affected by the electron mobility of graphene. This study may be applied to the initial design of a structure for active control, and facilitate the development of graphene-based thermal devices.

## Figures and Tables

**Table 1:** Design parameters and obtained quality factors of the graphene ribbon metasurface.

Target Wavelength	*λ*_t_ [µm]	6	8	10
Period	Λ [µm]	2.85	4.01	5.23
Width of graphene ribbon	*W* [µm]	0.134	0.231	0.355
Thickness of dielectric	*d* [µm]	0.891	1.25	1.64
Relaxation time of graphene	*τ* [ps]	0.329	0.296	0.273
Fermi energy of graphene	*E*_F_ [eV]	1.64	1.48	1.37
Quality factor	Q	56.0	40.0	25.9

**Table 2:** Design parameters and obtained quality factors of graphene ribbon metasurface used for the calculation in Figure 7a.

Mobility of Graphene	*µ* [m^2^Vs]	0.10	0.20	0.60	2.0	6.0
Target wavelength	*λ*_t_ [µm]	10				
Period	Λ [µm]	5.24	5.23	5.24	5.24	5.23
Width of graphene ribbon	*W* [µm]	0.447	0.355	0.246	0.165	0.114
Thickness of dielectric	*d* [µm]	1.64				
Relaxation time of graphene	*τ* [ps]	0.172	0.237	0.569	1.27	2.64
Fermi energy of graphene	*E*_F_ [eV]	1.72	1.37	0.948	0.635	0.440
Quality factor	Q	15.5	25.9	52.2	114	220
